# Immunosuppressive and Non‐Immunosuppressive Drugs for Heart Xenotransplantation in Humans: Is Europe Ready?

**DOI:** 10.1111/xen.70061

**Published:** 2025-06-30

**Authors:** Nicola Pradegan, Annamaria Tolomeo, Diego Ciccarelli, Emanuele Cozzi, Antonio Vitiello, Andrea Zovi, Gino Gerosa

**Affiliations:** ^1^ Cardiac Surgery Unit and Heart Transplant Program Padova University Hospital Padua Italy; ^2^ Department of Cardiac Thoracic and Vascular Science and Public Health Cardiovascular Regenerative Medicine Lab, University of Padova Padua Italy; ^3^ Institute of Pediatric Research “Città della Speranza” Padua Italy; ^4^ Department of Cardiac Thoracic and Vascular Science and Public Health Transplant Immunology Lab, University of Padova Padua Italy; ^5^ Transplant Immunology Unit Padova University Hospital Padua Italy; ^6^ Pharmacy Department National Health Ministry Rome Italy

**Keywords:** Europe, heart xenotransplantation, immunosuppression therapy, medicines agency

## Abstract

Xenotransplantation is becoming an emerging field of interest for the treatment of end‐stage heart disease. In fact, the shortage of human heart donors in European countries requires the increasing investigation of alternative strategies such as heart xenotransplantation. Among different limitations in this peculiar field, the experimental pharmacological management of patients who undergo heart xenotransplantation is primarily narrowed by the indications for which these drugs have been previously authorized by international medicines agencies. In fact, many of these medications have been never authorized for transplant rejection therapy, or for xenotransplantation, but their mechanism of action might stop the molecular pathways which are involved in xenograft antibody‐mediated rejection. Additionally, drugs costs and supply can restrict their availability for xenotransplantation practice. The aim of this review is to describe the current drugs which have been used in the clinical cases of heart xenotransplantation performed to date, to analyze the indications for which they are authorized, and to evaluate the future medications which might be implemented in heart xenotransplantation field, with a particular focus on the European scenario.

## 1 Introduction

Even if patients requiring heart transplantation (HT) for advanced heart failure are increasing, the current available amount of human heart donors is limited for supplying the real demand for organ transplantation [1]. Consequently, researchers are looking for alternative therapeutic options such as total artificial hearts or bio‐engineered grafts. Xenotransplantation represents an emergent therapy [2, 3]: early experience with the use of genetically modified pig hearts in non‐human primates (NHP) [4, 5, 6, 7] and in humans seems to be promising [8, 9, 10, 11], even if different concerns regarding organ immunotolerance still must be addressed. In addition to a complex animal gene‐editing to reduce the rate of graft rejection [12], the human recipient requires an in intensive immunosuppressive drug regimen in order to lower the cellular and antibody‐mediated immunoreaction [13]. Differently from allotransplantation, in xenotransplantation practice, innovative drugs have been added to the conventional immunosuppressive pharmacological therapy in order to hit the B cells and antibody‐mediated immune reaction. However, many of these drugs are still under investigation, or are not primarily indicated for immunosuppressive use, or are not available in Europe. The aim of this review is to analyze the pharmacological properties of the drugs used in current heart xenotransplantation clinical cases, to investigate the current European indications and limits in their use, and in the future perspectives in this field. We think that a similar review might help regulatory agencies in understanding how to use these drugs in the context of setting a clinical heart xenotransplantation program in European countries.

## 2 Drugs Used for Clinical 10‐Gene Edited (GE) Heart Xenotransplantation

### 2.1 Conventional Drugs

#### 2.1.1 Rabbit Antithymocyte Globulin (rATG)

rATGs are potent lymphocyte T‐depleting antibodies which are used as part of the induction therapy in human allotransplantation. This drug is approved for clinical use in the European Union (E.U.) and it is indicated for the prophylaxis and treatment of rejection episodes after HT [14]. The main mechanism of action involves interaction with cell surface markers of immune cells, with immunosuppressive effects. Although the predominant mechanism involves T‐cell depletion via the complement‐mediated pathway, alternative mechanisms have been elucidated. The route of administration is intravenous. Even if current guidelines still debate on the effective benefit of this strategy in reducing acute cardiac graft rejection [15], the more recent literature suggests rATG superiority in preventing immunoreactions after heart allotransplantation [16]. Since rATG have been used in NHP undergoing orthotopic HT, rATG induction was used in the first published human xenotransplantation similarly to the conventional protocol used with human hearts [8]. This drug was also used as induction treatment in two pilot brain‐dead cases [10].

#### 2.1.2 Mycophenolate Mofetil (MMF)

MMF is a prodrug of mycophenolic acid. It is authorized in the E.U. for the prophylaxis of acute rejection in patients receiving renal, cardiac, or liver transplantation. It is a potent, selective, non‐competitive, and reversible inhibitor of inosine monophosphate dehydrogenase (IMPDH), and thus inhibits de novo synthesis of the guanosine nucleotide without being incorporated into DNA. Since the proliferation of T and B lymphocytes is critically dependent on de novo synthesis of purines, where other cell types can use the purine reuse mechanism, MMF exerts a more potent cytostatic effect on lymphocytes than on other cells.

Besides its inhibition of IMPDH and subsequent deprivation of lymphocytes, MMF also affects the cellular checkpoints responsible for lymphocyte metabolic programming. It has been shown, using human CD4+ T cells, that MMF shifts transcriptional activities in lymphocytes from a proliferative state to catabolic processes relevant to metabolism and survival, leading to an anergic T‐cell state, in which cells become irresponsive. The route of administration is oral. Current International Society of Heart and Lung Transplantation (ISHLT) guidelines recommend MMF use (in addition to calcineurin inhibitor‐based therapy) because this combination have been shown to reduce onset and progression of cardiac allograft vasculopathy (CAV) [15]. MMF has been also used within the first 3 weeks after the first heart xenotransplant, and then for additional other 7 days in the last 10 days of life of the first xenograft recipient [8]. Indeed, MMF was administered in the two brain‐dead cases who received the 10‐gene edited xenografts [10].

#### 2.1.3 Tacrolimus

Tacrolimus is a calcineurin inhibitor, and it inhibits T‐cell proliferation by binding to FK506 binding protein. Evidence shows that tacrolimus inhibits T‐lymphocyte activation and T‐helper‐dependent B‐lymphocyte proliferation, as well as the production of lymphokines (such as interleukin‐2, interleukin‐3, and γ‐interferon), and interleukin‐2 receptor expression. The route of administration is oral. Tacrolimus has been approved by European Medicines Agency (EMA) in the prevention and treatment of solid‐organ transplant rejection. This drug is widely used in heart allotransplantation, alone or in combination with other immunosuppressive drugs [17], and it is recommended as part of the maintenance regimen. In the first human cardiac xenotransplantation, tacrolimus was part of the immunosuppressive therapy.

#### 2.1.4 Steroids

Corticosteroids (CS) are commonly used anti‐inflammatory medicines which lower inflammation and reduce immune system activity. Their molecules bind to glucocorticoid receptor, inhibiting pro‐inflammatory signals, and promoting anti‐inflammatory signals [18]. For their nonspecific mechanism of action and their broad spectrum of side effects, they should be preferentially used in the first period after transplantation; indeed, a rapid withdrawal or minimization within 3–12 months should be achieved and it is recommended to limit long‐term CS side effects. Steroids utilization in heart xenotransplantation was also reported: in particular, in the first case, after a pulse dose of methylprednisolone (1000 mg/day of xenotransplant), maintenance immunosuppression included a rapid methylprednisolone taper (125–30 mg daily) [8]. Induction therapy with steroids was also used in the brain‐deceased cases of xenotransplantation [10].

#### 2.1.5 Antivirals

*Ganciclovir* is a DNA polymerase inhibitor used to treat cytomegalovirus (CMV) and herpetic keratitis of the eye. In particular, this drug is indicated for induction and maintenance in the treatment of CMV retinitis in immunocompromised patients, including patients with acquired immunodeficiency syndrome (AIDS). It is also used in the treatment of severe CMV disease, including CMV pneumonia, CMV gastrointestinal disease, and disseminated CMV infections, in immunocompromised patients. Ganciclovir, approved both by FDA and EMA, is preferentially administered intravenously and it is routinely prescribed both for CMV prophylaxis or treatment in HT recipients (according to donor serological status). This medication was used prophylactically in the first Baltimore case recipient since the patient was human‐CMV IgG negative. Due to leukopenia, the patient was shifted to *Valaciclovir* for 11 days. *Valaciclovir*, an old drug approved by FDA in 1995 for herpes infections and by EMA in 2010, is indicated for CMV prophylaxis and/or treatment in solid organ recipients.

### 2.2 Unconventional Off‐Label HT Recommended Drugs

#### 2.2.1 Eculizumab

Eculizumab (Soliris) is a monoclonal antibody which is recommended by current ISHLT Guidelines for hyperacute and acute antibody‐mediated rejection (AMR) after heart allotransplantation [15]. This drug targets complement protein C5, binding it and preventing the activation of a complement terminal complex. Food and Drug Administration (FDA) and EMA approvals arrived on 2007. Primary indication for eculizumab use in U.S. is paroxysmal nocturnal hemoglobinuria (PNH) to reduce hemolysis, atypical hemolytic uremic syndrome to inhibit complement‐mediated thrombotic microangiopathy, and neuromyelitis optica spectrum disorder. In E.U. it is approved for PNH, for atypical hemolytic uremic syndrome and for refractory generalized myasthenia gravis. Consequently, its use in HT AMR treatment, even if recommended, is off‐label. In the first xenotransplantation case, eculizumab 1200 mg was used only at an advanced stage when AMR was diagnosed, in combination to other drugs [8]. In the two brain‐dead donor cases by New York University (NYU), eculizumab was used as induction therapy on postoperative day (POD) #0 (1200 mg) and POD#1 (900 mg) [10]. In the second Baltimore heart xenotransplantation, eculizumab was used on POD#16 after the histopathological evidence of AMR at the first post‐operative biopsy.

#### 2.2.2 Rituximab

Rituximab (MabThera) is also a monoclonal antibody that targets CD20. Rituximab binds specifically to the transmembrane antigen CD20, a non‐glycosylated phosphoprotein found on pre‐B lymphocytes and mature B lymphocytes. This drug was originally approved by FDA in 1997 as a single agent to treat patients with B‐cell Non‐Hodgkin Lymphoma (NHL). Later, the drug was approved for additional neoplastic and autoimmune diseases (e.g., chronic lymphocytic leukemia, connective tissue disease). To date, EMA approved rituximab in NHL, chronic lymphocytic leukemia, rheumatoid arthritis, granulomatosis, and pemphigus vulgaris. The route of administration is intravenous. In the context of human HT, even if off‐label, rituximab has been used for the management of AMR or desensitization therapy, alone or in combination with other treatments [19, 20]. Rituximab has been used only in the first xenotransplantation protocol of Baltimore as induction therapy (POD #‐1), 1 week after transplantation, and at the time of AMR on POD #55 [8].

#### 2.2.3 Intravenous Immune globulin (IVIG)

IVIG (Privigen) is a purified form of human immunoglobulin G and other proteins used to treat immunodeficiency and a wide variety of autoimmune disorders. IVIG contains the same distribution of IgG antibody subclasses as is found in the general human population. IVIG is immediately and completely bioavailable in the recipient's circulation after intravenous administration. It is distributed relatively rapidly between plasma and extravascular fluid. IVIG is indicated and approved for primary immunodeficiency and several other immune‐mediated diseases (e.g., immune thrombocytopenic purpura, chronic inflammatory demyelinating polyneuropathy, multifocal motor neuropathy, Kawasaki Syndrome, etc.). Its use in HT recipients is advised according to most recent guidelines for removing circulating antibodies or decrease their reactivity (Class IIa, Level of Evidence C). In the first heart xenotransplantation case, IVIG were prescribed for hypogammaglobulinaemia and during the first plasma exchange. However, according to laboratory tests, IVIG bound strongly to donor endothelium, possibly causing immune activation and consequent immune‐mediated graft failure leading to death of the patient. Consequently, they were not used in the second Baltimore case (despite multiple plasma exchange procedures).

### 2.3 Off‐Label HT No‐Recommended Drugs

#### 2.3.1 C1 Esterase Inhibitor (Anti‐C1, C1‐INH)

C1‐INH (Berinert, King of Prussia, PA, USA) is a C1 esterase inhibitor and consists of purified endogenous complement component‐1 esterase inhibitor (hC1INH), a molecule which regulates the activation of the complement and contact system pathways. This drug is approved in the U.S. and in the E.U. for the prophylaxis and treatment of hereditary angioedema. Its use was first explored in kidney transplantation to prevent or reduce hyperacute rejection, with promising preliminary in vitro results [21]. Furthermore, early clinical studies have demonstrated beneficial effects with minimal toxicity in lowering acute AMR [22, 23]. Its use is not approved or documented in human HT, while pre‐clinical experimentation in NHP receiving pig hearts [24] has moved researchers toward its use in human xenotransplantation: in fact, complement activation is a major issue in xenotransplant acute rejection, and C1‐INH, differently from other anti‐complement drugs (e.g., eculizumab) acts proximally in the complement cascade and consequently might show a more efficient effect in turning down the rejection process [25]. Berinert was used as induction and maintenance immunosuppressive therapy in the Baltimore case (10 mg/kg), using repeated single doses according to previous experience in NHP. This drug was also used in the second Baltimore case [11].

#### 2.3.2 KPL‐404 (Anti‐CD40)

KPL‐404 (Kiniksa Pharmaceuticals, Ltd., Hamilton, Bermuda) is a humanized IgG4 monoclonal antibody which is engineered to bind CD40 and interfere with CD40–CD40L interaction, and downstream T‐cell–dependent B‐cell–immune responses without triggering significant B‐cell depletion or Fc effector functions. This drug has not yet been approved by FDA or EMA, and it is at an experimental level: in particular, data on a phase 1 randomized clinical trial in healthy subjects have demonstrated its safety and efficacy [26]. Preliminary data on patients with rheumatoid arthritis have shown promising favorable clinical and laboratory effects [27]. This drug was part of the first Baltimore case, as induction and maintenance therapy [8].

#### 2.3.3 Tegoprubart (Anti‐CD40 Ligand–Anti‐CD40L)

Tegoprubart (formerly known as AT‐1501, Eledon Pharmaceuticals, Inc., Irvine, CA, USA) is a humanized Anti‐CD40L monoclonal antibody which addresses the central role of CD40L signaling in both adaptive and innate immune cell activation. This drug has not been approved by FDA and EMA yet, but several studies are investigating its role in kidney allograft transplantation, xenotransplantation, and amyotrophic lateral sclerosis (ALS). In particular, tegoprubart has been evaluated in a Phase 2A clinical trial on the safety and tolerability of this drug in ALS [28], while its applicability in islet and kidney allotransplantation is still at a pre‐clinical phase [29]. Within the field of xenotransplantation, tegoprubart was used as induction and maintenance therapy in the second human heart xenotransplant case performed by the Baltimore group [11] instead of an anti‐CD40 agent (initial dose = 20 mg/kg IV with weekly dosing adjusted by pharmacokinetic modeling for a goal level of 600–1000 mcg/mL). Tegoprubart was specifically used in the second case because of its costimulation blockade mechanism (which seems to be a promising target in xenotransplantation immunosuppressive pharmacology). Unfortunately, despite this innovative therapy, authors reported early AMR even in this second human case: in particular, authors state that AMR was detected at the first endomyocardial biopsy 13 days after transplantation, while therapeutic tegoprubart blood levels were not attained until POD #8 [11]. Consequently, this window of subtherapeutic tegoprubart allows the possibility that graft failure may have resulted from a lapse in costimulation blockade. Strict pharmacokinetic surveillance of tegoprubart might have had a role in preventing AMR development early after transplantation.

#### 2.3.4 Carfilzomib (Kyprolis)

Carfilzomib is a proteasome inhibitor used either alone or in conjunction with a chemotherapy regimen to treat patients with relapsed or refractory multiple myeloma. This medication, approved by FDA and by EMA in 2012 and 2015, respectively, has been used off‐label in the transplantation field: in particular, it was successfully used in a small cohort of sensitized HT candidates to reduce the level of Human Leukocyte Antigen (HLA) antibodies and complement binding [30]; in the pre‐clinical scenario, it was used in a non‐human primate model of antibody‐mediated rejection in kidney transplantation [31]; in clinical practice, emerging evidence is suggesting its favorable use in AMR after pediatric kidney transplantation [32] and after lung transplantation [33]. Carfilzomib was used in the second Baltimore case (27 mg m^−2^) to suppress the potential plasma cell source of anti‐porcine antibody production.

#### 2.3.5 Antivirals

*Cidofovir (Vistide)* is an injectable antiviral medication employed in the treatment of CMV retinitis in patients diagnosed with acquired immunodeficiency syndrome. The major route of elimination of cidofovir was found to be renal excretion of the unmetabolized drug, through a combination of glomerular filtration and tubular secretion. In vitro, the protein binding of cidofovir to plasma or serum proteins was 10% or less over a cidofovir concentration range of 0.25–25 µg/mL. Its off‐label use in HT recipients is widely describes [34, 35, 36]. The Baltimore group used this drug in the first xenograft recipient when porcine CMV (PCMV) microbial cell‐free DNA titers started to rise. This choice was based to the evidence that cidofovir is a valid agent against PCMV according to previous literature [37, 38].

## 3 New Drugs

### 3.1 Additional Anti‐Complement Medications

Complement system activation is a key effector mechanism underlying AMR, and current pharmacological regimens used in heart xenotransplantation have used drugs against the complement cascade. However, current therapies might not be sufficient in preventing acute immune reactions. Among new anti‐complement therapies, *Ravulizumab (Ultomiris)* is a potent and selective C5 inhibitor, approved by FDA and EMA; it is indicated for the treatment of PNH, atypical haemolytic uremic syndrome, generalized myasthenia gravis and Neuromyelitis optica spectrum disorder. Ravulizumab is an IgG2/4k monoclonal antibody that specifically binds to the complement protein C5, thereby inhibiting its cleavage at C5a (the proinflammatory anaphyllotoxin) and C5b (the subunit that initiates the membrane attack complex [MAC or C5b‐9]) and preventing the formation of the C5b‐9 complex for the treatment of PNH and atypical hemolytic uremic syndrome in children and adults. The route of administration is intravenous. It was engineered from eculizumab to increase the duration of action and reduce the frequency of drug administration. In solid organ transplantation, ravulizumab has been used after kidney transplant to treat or prevent atypical hemolytic uremic syndrome, but there are no reports on its use for treating or preventing any immune reaction [39, 40]. *Pegcetacoplan (Aspaveli)* is a new anti C3 drug, approved both in U.S. and E.U. in monotherapy in adults to treat PNH with anemia, similarly to anti C5 eculizumab. In transplantation, this drug has been tested in kidney transplant recipients who developed post‐transplant recurrent C3 glomerulopathy or immune complex membranoproliferative glomerulonephritis (NOBLE study) [41]: this preliminary trial has shown a significant reduction of C3 activation (60%) in the enrolled patients. *Sutimlimab (Enjaymo)* is a proximal classical complement C1s inhibitor which is indicated for the treatment of hemolysis in adults with cold agglutinin disease. This drug has been approved by FDA and EMA for this indication after the publication of the CADENZA trial [42], which has shown a near‐complete inhibition of the classical complement pathway in the experimental group of patients. Unfortunately, no literature is available about its use in the transplantation practice.

### 3.2 Additional Anti‐AMR Drugs

AMR can be targeted at different levels of the physiopathological mechanisms which underly this complication (e.g., B cells, plasma cells, Interleukin 6, circulating pathogenic antibodies). *Alemtuzumab (Campath, Lemtrada, MabCampath)* is a monoclonal antibody directed toward the lymphocyte antigen CD52. Alemtuzumab was approved by FDA in 2001. It is marketed for multiple sclerosis treatment and for B‐cell chronic lymphocytic leukemia. EMA approved it in 2001 for the treatment of patients with B‐cell chronic lymphocytic leukemia. This drug has been studied in randomized controlled trials and has demonstrated low levels of rejection in renal transplant recipients compared with other induction agents, with favorable outcomes at 12 and 18 months post‐transplant [43, 44, 45]. Indeed, it has been efficiently used in lung transplant recipients [46], as well as in the HT induction strategy [47]. However, its effect on AMR treatment after HT has shown discordant results, especially in the pediatric population [48, 49]. *Bortezomib (Velcade)* is a proteasome inhibitor used to treat multiple myeloma in subjects who are refractory to previous first line therapies, and it is also approved for mantle cell lymphoma. FDA approved it in 2003 as the first anticancer proteasome inhibitor. EMA approved it the following year for the same indication. Bortezomib has shown promising effects both in kidney [50] and lung [51] transplantation AMR mitigation, but data are still insufficient for its broad application in this field. Its application in desensitization protocols before HT has been widely reported with variable results [52, 53, 54]; however, no reports are available on AMR management after HT. *Daratumumab* ( *Darzalex* ) is a human IgG1κ monoclonal antibody (mAb) that binds to the CD38 protein expressed on the surface of cells in a variety of hematological malignancies, including clonal plasma cells in multiple myeloma and AL amyloidosis, as well as in other cell and tissue types. The CD38 protein has multiple functions, such as receptor‐mediated adhesion, signal transduction activity and enzyme activity. The drug is approved either by FDA (2015) and EMA (2016) for multiple myeloma treatment. Its application in solid organ transplantation is still anecdotic (two reports after human kidney transplantation and history of multiple myeloma [55] or amyloidosis [56], respectively, showing a reduction in cells which are usually involved in AMR). It has been also successfully reported in a case of AMR after HT [57]. Another drug directed toward CD38 protein is *Felzartamab*: this medication, still under investigation, has been recently recognized as an emerging target for the treatment of late AMR without T‐cell mediated rejection in kidney transplant patients, with promising early results in phase 2, double‐blind, randomized, placebo‐controlled trial [58]. *Tocilizumab (Actemra, RoActemra, Tofidence)* is a recombinant humanized monoclonal antibody IL‐6 receptor inhibitor used to treat inflammatory and autoimmune conditions. It was granted FDA approval in 2010 to treat different types of arthritis and cytokine release syndrome. By December 2021 EMA approved it also for COVID‐19 management. Its clinical application in AMR management in solid organ transplantation has been successfully described both for kidney [59] and lung transplant [60]. For the heart, even if used in several HT recipients for COVID‐19 therapy [61, 62], its application in AMR therapy is still limited to a very small group of patients [63]. Unfortunately, tocilizumab use in pig‐to‐baboon organ xenotransplantation has shown preliminary detrimental effects within in vitro observations [64]. *Imlifidase ( Idefirix)* is a cysteine protease that specifically cleaves human IgG antibodies, facilitating kidney transplantation in HLA sensitized patients [65]. Consequently (as approved by EMA in 2020) it is indicated for desensitization of highly sensitized adult kidney transplant recipients with a positive crossmatch against an available deceased donor. No reports are available on Imlifidase use in human HT. A preliminary study in a sensitized non‐human primate found a significant reduction in anti‐pig antibodies after Imlifidase administration, opening a window on its use in xenotransplantation. However, given its high immunogenicity, the drug can be used only once, thus significantly limiting its durability as monotherapy.

### 3.3 Costimulation Blockade (Belatacept, Anti‐CD40L/CD154)

Costimulation blockade has been shown as an effective way to suppress the immune response in transplantation against donor antigens and to prolong graft survival. In particular, CD80/CD86 and CD40/CD40L[CD154] pathways play a pivotal role in transplantation and have been used to create specific pharmacological targets. *Belatacept (Nulojix*) is a high‐affinity CTLA4‐Ig variant, approved by FDA and EMA in 2011 and indicated for prophylaxis of graft rejection in adults receiving a kidney transplant. Data on the utilization of *Belatacept* therapy in heart transplant (HT) recipients are scarce. In particular it specifically binds to CD80 and CD86 receptors that are found on the antigen‐presenting cell (B cells, macrophages, dendritic cells) to block selective T‐cell lymphocyte costimulation. The reconstituted solution of *Belatacept* should be administered as an intravenous infusion at a relatively constant rate over 30 min. Current trials (BENEFIT and BENEFIT‐EXT) demonstrate its efficacy in kidney transplantation immunosuppression therapy [66], but its application in other solid organ transplantation fields (e.g., pediatric transplantation, lung, liver transplantation, AMR) are still limited to small series. Belatacept use in HT is limited to a retrospective single center study showing the non‐inferiority of this therapy in terms of post‐transplant rejections [67]. In xenotransplantation, this drug has been under‐investigated and current evidences are still at an in vitro phase, showing promising and beneficial effect on xenoreactivity [68, 69]. *Anti‐CD40L/CD154*, which includes different types of antibodies directed toward CD154 (e.g., ruplizumab, toralizumab, ABI793, H106, dapirolizumab pegol, letolizumab, dazodalibep, TNX‐1500) has shown great promise in rodent and NHP models for inhibiting rejection and prolonging graft survival. Treatment with anti‐CD154 antibody has effectively reduced donor‐specific antibody (DSA) production in these models. Costimulation blockade by anti‐CD154 antibody prolongs graft survival, but its wide use in the experimental and clinical setting was initially limited because of thromboembolic complications [70]; however, more recent and smaller molecules are free of thromboembolic events. In particular, TNX‐1500 has demonstrated to be well tolerated, to prolong allograft survival, and prevent alloantibody production and CAV in a stringent preclinical non‐human primate heart allotransplant model [71] as well as in a non‐human primate renal allograft experiment [72]. Combined CD40+CD40L blockade in xenotransplantation has been recently experimented in six NHP models by combining a chimeric anti‐CD40 antibody with an investigational long‐acting PASylated anti‐CD40L Fab fragment with promising results (non‐inferior survival, no thromboembolic events) [73].

Table 1 summarizes the main properties, indications, and current knowledge on the use of the previous off‐label drugs within heart allotransplantation and xenotransplantation.

**TABLE 1:** Summarize of the off‐label drugs used in clinical experimental heart xenotransplantation and other promising medications which might get future involvement in the field, with a focus on their indications and current available literature of applicability within the field of heart transplantation.

Drug name (Commercial brand)	FDA/EMA approved and year of approval	Clinical indications	Route of administration	Studies in heart allotransplantation	Studies in heart xenotransplantation
Eculizumab (Soliris)	FDA 2007 EMA 2007	Treatment of paroxysmal nocturnal hemoglobinuria, atypical hemolytic uremic syndrome, neuromyelitis optica spectrum disorder	IV	Off‐label use for AMR treatment	Used in the Baltimore xenotransplantation cases at an advanced stage and in the two dead‐brain donor cases at New York University as induction therapy
Rituximab (MabThera)	FDA 1997 EMA 1998	Treatment of non‐Hodgkin lymphoma, chronic lymphocytic leukemia, rheumatoid arthritis, granulomatosis with polyangiitis, microscopic polyangiitis, pemphigus vulgaris	IV	Off‐label use for the management of AMR or desensitization therapy	Used in the protocol of Baltimore as induction therapy and when AMR occurred
IVIG (Privigen)	FDA 2007 EMA 2008	Primary immunodeficiency and several other immune‐mediated diseases (e.g., immune thrombocytopenic purpura, chronic inflammatory demyelinating polyneuropathy, multifocal motor neuropathy, Kawasaki Syndrome, etc)	IV	Off‐label use for AMR therapy (antibodies removal, antibody reactivity reduction)	Used in the first Baltimore case to treat hypogammaglobulinemia and during the first plasma exchange
C1 Esterase Inhibitor (Berinert)	FDA 2009 EMA 2013	Prophylaxis and treatment of hereditary angioedema	IV, SC	None	Used as induction and maintenance therapy in the Baltimore cases
KPL‐404 (anti‐CD40)	Not approved yet	Undergoing evaluation for the treatment of autoimmune diseases	IV, SC	None	Used as induction and maintenance therapy in the first Baltimore case
Tegoprubart (anti‐CD40L)	Not approved yet	Undergoing evaluation for the treatment of amyotrophic lateral sclerosis	IV	None	Used as induction and maintenance therapy in the second Baltimore case
Carfilzomib	FDA 2012 EMA 2015	Alone or in conjunction with a chemotherapy regimen to treat patients with relapsed or refractory multiple myeloma	IV	Off‐label use in sensitized HT candidates to reduce the level of HLA antibodies and complement binding	Used for AMR treatment in the second Baltimore case
Cidofovir	FDA 1996 EMA 1997 (withdrawn in 2015)	Citomegalovirus retinits treatment in adults with acquired immunodeficiency syndrome	IV	None	Porcine cytomegalovirus activation treatment in the first Baltimore case
Ravulizumab (Ultomiris)	FDA 2018 EMA 2019	Treatment of paroxysmal nocturnal hemoglobinuria, atypical hemolytic uremic syndrome, generalized myasthenia gravis in adults, neuromyelitis optica spectrum disorder	IV	None	None
Pegcetacoplan (Aspaveli)	FDA 2021 EMA 2021	Treatment of paroxysmal nocturnal hemoglobinuria with anemia in adults	SC	None	None
Sutimlimab (Enjaymo)	FDA 2022 EMA 2022	Treatment of hemolysis in adults with cold agglutinin disease	IV	None	None
Alemtuzumab (Campath, Lemtrada, MabCampath)	FDA 2001 EMA 2001	Treatment of multiple sclerosis, B‐cell chronic lymphocytic leukemia	IV	Used for the treatment of AMR	None
Bortezomib (Velcade)	FDA 2003 EMA 2004	Treatment of multiple myeloma, mantle cell lymphoma	IV, SC	Used in desensitization protocols	None
Daratumumab (Darzalex)	FDA 2015 EMA 2016	Treatment of multiple myeloma	IV	Used in a case of AMR	None
Felzartamab	Undergoing clinical studies	Membranous Nephropathy treatment Late AMR no T‐cell mediated treatment in kidney transplant	IV	None	None
Tocilizumab (Actemra, RoActemra, Tofidence)	FDA 2010 EMA 2009	Treatment of rheumatoid arthritis, giant cell arteritis, juvenile idiopathic arthritis, systemic sclerosis‐associated interstitial lung disease, cytokine release syndrome, COVID‐19	IV	Used for the management of AMR	In vitro
Imlifidase (Idefirix)	FDA not approved EMA 2020	Desensitization of highly sensitized adult kidney transplant recipients	IV	None	Preliminary study in a non‐human primate
Belatacept (Nulojix)	FDA 2011 EMA 2011	Prophylaxis of organ rejection in kidney transplant	IV	Retrospective clinical small series on post‐transplant rejection outcomes	In vitro
Anti‐CD40L/CD154, that is, ruplizumab, toralizumab, ABI793, H106, dapirolizumab pegol, letolizumab, dazodalibep, TNX‐1500	Undergoing clinical studies	Promising for inhibition of rejection and prolongation of graft survival	—	TNX‐1500: pre‐clinical non‐human primate cardiac allograft model (*n* = 10)	Combined CD40+CD40L blockade study in six non‐human primates

Abbreviations: AMR, antibody‐mediated rejection; EMA, European Medicines Agencies; FDA, Food and Drug Administration; HLA, human leukocyte antigen; IV, intravenous; SC, subcutaneous.

## 4 EU Limits to Current Used Drugs

Even though new evidence produced in the U.S., currently in the E.U. there has not been acceleration by regulatory authorities to expand the indications for drugs already recommended for the treatment of AMR in patients undergoing HT or in view of xenotransplantation, and this affects also those medications which are used for microbiological prophylaxis or treatment. Unfortunately, this is in contrast with the active role of several European research groups in the field of xenotransplantation [2, 74]. In fact, all drugs considered by international guidelines for transplantation and xenotransplantation as unconventional or new, are used under an off‐label regimen. As a result, this indication is currently never listed in the package leaflets of these medicines, therefore the procedures that clinicians have to follow to prescribe these medicines may be hard to apply and can discourage their applications in the clinical setting. On the basis of the evidence presented, this review aimed to bring to the attention of the scientific community and medicines regulatory authorities that, although preliminary, these therapies could be considered promising in the indication of the prophylaxis of AMR or viral infections in patients undergoing xenotransplantation in a near future. It is desirable that the generation of new evidence through real case‐reports can produce more strong evidence to expand the indications for the use of non‐conventional and new drugs listed by international guidelines and summarized in Table 1. The purpose of the scientific community must be the increase of the awareness of the use of this practice and the stimulation of its application in the clinical settings. Expanding the therapeutic armamentarium available for the prophylaxis of acute rejection and human and/or pig viral infections in heart transplant patients could enable science to make great strides and save additional lives, and improve the quality of life of transplanted organ recipients.

## 5 Conclusion

Heart xenotransplantation is surging as a new alternative strategy to treat patients with advanced heart failure. Current evidences in pre‐clinical studies have shown promising results in terms of immune tolerability and graft durability; however, preliminary clinical results have not demonstrated mid‐term favorable and reproducible results. Within the limitations of current xenotransplantation practice, drugs provision represents a main issue for the onset of future clinical trials. In fact, many of the drugs which have been implemented in heart xenotransplantation were tested in pre‐clinical scenarios and were not applied to specific clinical trials on heart xenotransplantation rejection. Consequently, they are still under investigation or, if authorized by international organizations (e.g., FDA, EMA), these drugs are off‐label. Besides, the expensive costs of many of these products require a careful evaluation of the risk/benefit ratio. Finally, the fact that many of the drugs already used and/or approved by the FDA are not yet approved by European authorities limits the application of existing experimental protocols in a potential European scenario. Consequently, before clinical implementation of xenotransplantation in the care of end‐stage heart disease, a deep communication between drug authorization agencies, national health authorities and university hospitals with xenotransplantation programs is mandatory.

## Conflicts of Interest

The authors declare no conflicts of interest.
